# Adolescent E-cigarette or Vaping Use-Associated Lung Injury in the Delaware Valley: A Review of Hospital-Based Presentation, Management, and Outcomes

**DOI:** 10.7759/cureus.21988

**Published:** 2022-02-07

**Authors:** Sarah Schaffer, Abigail Strang, David Saul, Vijay Krishnan, Aaron Chidekel

**Affiliations:** 1 Pulmonology, Nemours Children's Hospital, Delaware, Wilmington, USA; 2 Radiology, Nemours Children's Hospital, Delaware, Wilmington, USA; 3 Radiology, University of Maryland Medical Center, Baltimore, USA

**Keywords:** acute lung injury, adolescent cannabis use, adolescent nicotine use, vaping, e-cigarette

## Abstract

E-cigarette or vaping use-associated lung injury (EVALI) remains a major concern due to ongoing use of nicotine and/or cannabis-containing products and resulting acute lung injury. There are few published reports describing the clinical features, comorbidities, severity of disease, and outcomes of treatment in adolescents. This report describes the experience of a single tertiary care children’s hospital in the Delaware Valley and reviews data from all patients diagnosed with EVALI in the emergency department and inpatient setting from July 2019 to June 2021 at the Nemours Children’s Hospital in Wilmington, Delaware. Demographic, clinical, therapeutic, diagnostic features, and outcomes are presented. Abstinence and steroids improved outcomes in our population. Obtaining a vaping history, negative infectious testing, elevated inflammatory markers, and characteristic computed tomography findings were key to making the diagnosis of EVALI.

## Introduction

The Centers for Disease Control and Prevention (CDC) defines e-cigarette or vaping product use-associated lung injury (EVALI) as an acute or subacute respiratory illness that can be severe and life-threatening. E-cigarette/vaping-associated lung injury was first described in June 2019 with emergency department-related visits peaking in September 2019 [1]. Similarly, at Nemours Children’s Hospital of Delaware, there was a dramatic rise of cases in the summer of 2019. As of February 2020, there have been 2,807 EVALI cases and deaths reported to the CDC, 15% of whom were under the age of 18 years [2]. There have been 68 confirmed deaths since February 2020 when the CDC stopped collecting data after a decline in cases was noted and the COVID-19 pandemic began.

The EVALI outbreak coincides with a sharp increase in adolescent e-cigarette and vaping product use. E-cigarettes work by heating a liquid, called “vape,” that produces an aerosol, which is then inhaled into the lungs. Vape liquid contains various flavors including fruit, candy, dessert, and menthol that are particularly appealing to adolescents. In 2020, of the youth who reported e-cigarette usage, 82.9% used flavored varieties [3]. E-cigarettes are also advertised to target youth similarly to other tobacco-containing products like cigarettes. In 2016, about seven in 10 middle school and high school students (69.3%) said they had seen e-cigarette advertising [4]. This has led to nearly one in four high school students and one in 15 middle school students in the United States reporting use of any tobacco product in 2020. E-cigarettes were the most commonly used product, accounting for tobacco use in 19.6% of high school students and 4.7% of middle school students [5,6]. According to the 2017 Delaware Youth Risk Behavior Survey of public high school students conducted by the University of Delaware, 13.6% of students used e-cigarettes in the past month and 38% reported trying an electronic vapor product, which is significantly higher than the national average [7].

Adolescent EVALI presents with variable symptoms [8,9]. These range from constitutional symptoms to gastrointestinal, respiratory, and neurologic. Frequently, there are associated mental health comorbidities [9]. Chest radiographs can be variable. Some studies show that chest radiographs are routinely abnormal in EVALI, whereas some studies report normal and abnormal chest radiographs [8-10]. There have been few reports of pneumomediastinum, subcutaneous air, and/or pneumothorax [10]. Varying amounts of respiratory support are required in the acute stage of the disease, ranging from nasal cannula to intubation and mechanical ventilation [9]. Treatment with glucocorticoids often leads to improvement in symptoms, spirometry, and imaging [8,9,11].

There are few published reports describing the clinical features, comorbidities, severity of disease, and outcomes of treatment in adolescents. Characteristic features of EVALI in adolescent patients are limited as they are based on a few studies with small sample sizes. This study describes the experience of a single tertiary care children’s hospital in the Delaware Valley.

## Materials and methods

This study was reviewed and determined exempt by the Nemours Institutional Review Board. The electronic medical record (EMR) was queried to identify patients who met inclusion criteria (physician diagnosis of EVALI) who were treated at Nemours Children’s Hospital (Wilmington, Delaware) from July 2019 to June 2021. Patients were included in the case description if they were evaluated in the emergency room or required inpatient admission to the pediatric inpatient care unit and/or pediatric intensive care unit.

In addition to demographics (age, sex, race), the following past histories prior to EVALI diagnosis were reviewed: history of diagnosis of chronic respiratory condition (e.g., asthma); history of mental health diagnosis (e.g., attention deficit hyperactivity disorder, anxiety); and patient-reported use of vaping nicotine, tetrahydrocannabinol (THC), or both. Clinical symptoms and work-up during EVALI episode including presenting symptoms (weight loss, fever, cough, chest pain, shortness of breath, and gastrointestinal symptoms), prominent imaging findings (chest radiograph and chest computed tomography), laboratory findings (complete blood count, erythrocyte sedimentation rate, C-reactive protein, infectious testing, and urine drug screen), length of stay, highest level of respiratory support (nasal cannula, positive pressure, intubation, and extracorporeal membranous oxygenation), medical therapy (steroid use and nicotine replacement therapy), and lung function testing (pulmonary function testing and six-minute walk test) prior to discharge were reviewed.

Following the initial admission, the EMR was reviewed for a follow-up encounter with pulmonology and follow-up testing including imaging, laboratory testing, and pulmonary function testing. The EMR was used to identify any patients who were treated for a second episode of EVALI within the study timeframe. After data collection was complete, descriptive statistics were calculated to characterize features of the group.

## Results

Case descriptions

Twenty-five patients were diagnosed with EVALI during the study period. Most were males (64%), and the mean age was 16.5 years (range: 15-18 years). Nineteen patients were Caucasian, four were Hispanic, one was African American, and one was Asian. There was a remote history of asthma in four patients. The most common comorbidity was a mental health condition, including anxiety and depression in five patients, and anxiety alone in five patients. Fever and gastrointestinal symptoms were the most common presentation in over half the patients (n=14). Dyspnea was the most common presenting respiratory complaint (n=12), followed by cough (n=11) and chest pain (n=6). Significant weight loss was noted in eight patients on presentation. Both marijuana and nicotine use were endorsed in 12 patients, while 10 patients reported marijuana use only. Three patients reported only nicotine use, although two of these patients had urine drug screens positive for THC. The duration of vaping ranged from three months to three years. Sixteen patients had urine drug screens, and 15 were positive for THC. In addition to vaping, 4% of our patient population also reported smoking cigarettes (1/23), 39% (9/23) reported smoking marijuana using other methods (joints/bongs), 4% reported smoking hookah (1/23), and 52% (12/23) denied any other type of nicotine or marijuana inhalation than vaping.

Hospitalization was required in 24 patients who had an average length of stay of 7.8 days (range: 2-75 days). Most patients required respiratory support, which varied from intubation and extracorporeal membrane oxygenation (ECMO) for progressive respiratory failure (n=1), bilevel positive airway pressure (n=3), continuous positive airway pressure (n=1), high-flow nasal cannula (n=3) to standard nasal cannula (n=10).

**Figure 1:**
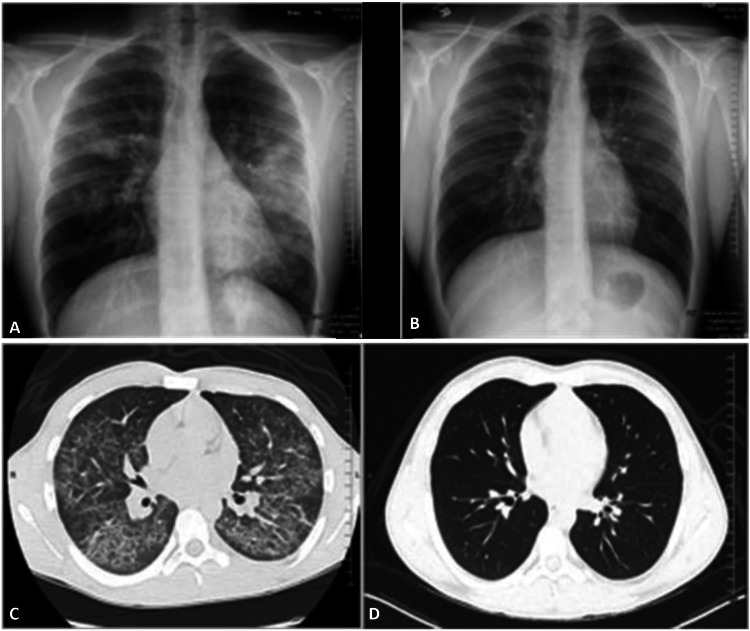
Chest Images A) Chest radiograph showing multifocal opacities, more on the left, including consolidation in the lingula, left lower lobe opacity, and right mid-lung field. B) Follow-up chest radiograph showing interval resolution of the previously noted bilateral air space opacities and mediastinal air. C) Chest computed tomography scan showing patchy areas of ground-glass opacification and interlobular septal thickening throughout both lungs with a lower lobe predominance and relative sparing of the periphery. D) Follow-up chest computed tomography scan showing near-complete resolution of the diffuse ground-glass opacification and interlobular septal thickening note on the prior study.

Chest radiographic findings were often bilateral, multifocal, and nonspecific. Two patients had a normal chest radiograph but showed abnormalities on chest computed tomography (CT). Chest computed tomography changes including multifocal, bilateral, and ground-glass opacities were noted in all patients, most severe in the dependent portions of the lung. Subpleural sparing was commonly described. Interstitial thickening was seen in almost all of the patients. Pneumomediastinum was seen in one patient. Lung consolidation was less frequent, usually occurring in more severe cases (Figures 1A-1D).

Respiratory viral panels (RVPs) were negative in 24 patients. One patient had a positive RVP for rhinovirus/enterovirus. Inflammatory markers (erythrocyte sedimentation rate/C-reactive protein) were elevated when measured (n=23). The average C-reactive protein value was 51.5 (range: 1.4-311), and the average erythrocyte sedimentation rate value was 66 (range: 7-116). Leukocytosis was seen in nearly all patients (n=23). Most hospitalized patients were treated initially with intravenous methylprednisolone (21/24) for an average of 4.3 days and transitioned to prednisone prior to discharge. The average duration of steroid taper was two weeks. Psychology consults were placed on 11 patients admitted to the hospital. One patient was treated with nicotine replacement therapy while intubated per the provider’s discretion. Six-minute walk tests were completed in 20 patients. Thirteen patients passed the test with normal oxygen saturations, whereas seven had abnormal results. Of the seven patients who failed the test, three passed a subsequent six-minute walk test prior to discharge and four were sent home with supplemental oxygen via nasal cannula. Spirometry was performed in hospitalized patients prior to discharge; eight showed mild airway obstruction, one showed moderate obstruction, one suggested mild restrictive lung disease, five showed mild obstruction suggesting restriction, and one showed normal spirometry. Diffusion capacity was also measured in 13 of these patients, which was reduced in six of these patients.

All patients seen for follow-up in the pulmonology clinic had improved symptoms with abstinence from vaping (n=21). There were 16 spirometry tests completed at follow-up, 15 of which were normal and one that showed mild restrictive lung disease. Diffusion capacity was decreased in four patients. Follow-up CT scans were completed in three patients, all of which showed improvement. Hospital readmission occurred in two patients; both reported vaping relapse. Results are summarized in Tables 1, 2.

**Table 1:** Patient Characteristics (n=25)

Variable	
Age, mean (range) years	16.5 (15-18)
Sex: % male/female	64/36
Ethnicity: Hispanic/non-Hispanic, number	4/21
Mental health diagnosis, number (%)	10 (41.7)
Prior pulmonary condition, number (%)	4 (16.7)
Reported marijuana use only, number	10
Reported nicotine use only, number	3
Reported both marijuana and nicotine use, number	12

**Table 2:** Clinical Course of Hospitalized Patients (n=24)

Variable	Number of Patients (% or Range)
Required hospital admission, number	24
Required respiratory support, number (%)	18/24 (75)
Nasal cannula	10
High-flow nasal cannula	3
Continuous positive airway pressure/bilevel positive airway pressure	4
Invasive mechanical ventilation and extracorporeal membrane oxygenation	1
Average length of stay, days (range)	7.8 (2-75)
Mean erythrocyte sedimentation rate/C-reactive protein (range)	66 (7-116)
Mean C-reactive protein (range)	51.5 (1.4-311)
Presence of leukocytosis (%)	23/24 (95.8)
Abnormal pulmonary function test (%)	15/16 (93.7)
Abnormal six-minute walk test, number (%)	13/20 (65)
Discharged with home oxygen, number (%)	4/24 (16.7)

## Discussion

Vaping continues to be a threat to the health of our adolescent population. As of February 2020, over 2,500 patients of all ages have been hospitalized with EVALI and there were 68 reported deaths, of whom 15% were younger than 18 years old [12]. The liquid in e-cigarettes can contain multiple substances including nicotine, THC, cannabinoid oils, flavorings, and additives that are known to be carcinogenic. The THC-containing vape products from informal sources are a major factor in the EVALI outbreak through product sampling and patient reports. Vitamin E acetate is a common additive in THC-containing vaping products and is believed to be a major contributor to the harmful effects of vaping. Vitamin E was found in 48 of 51 EVALI patients’ bronchoalveolar lavage samples in a recent study [13].

Tobacco product use is established primarily during adolescence, and vaping has been the most used tobacco product in this age group since 2014. Studies suggest that adolescents who vape are more likely to smoke cigarettes in the future, creating a perpetual cycle of nicotine addiction [14]. Although there was a decrease in e-cigarette use from 2019 to 2020, still 4.7% of middle school students and 19.6% of high school students report e-cigarette use in the past 30 days [4]. Most youth who report vaping use flavored e-cigarettes, most commonly fruit, menthol, and candy flavors. This, along with multiple other factors including sensitivity to nicotine, normalization through mass media, and accessibility, makes adolescents more likely to start and continue vaping. There is also a strong relationship between mental health issues, including anxiety, depression, and stress, and youth smoking [6]. There was a strong correlation in our subset of the population as well. Marijuana use in e-cigarette devices is also becoming increasingly more common. In 2016, one-third of middle and high school e-cigarette users reported vaping marijuana [1]. Action is being taken on a federal, state, and local level to reduce the youth vaping epidemic. In 2019, legislation was signed raising the federal minimum age of sale of tobacco products from 18 to 21 years. The Food and Drug Administration prohibited the sale of flavored rechargeable pods and cartridges in February 2020. Additional strategies including counter tobacco advertisement in the media, increased price policies, prohibiting the sale of disposable flavored products, and more programs that encourage smoke-free environments and lifestyles are still needed.

The presenting symptoms of our adolescent EVALI patients were broad and variable, with fever and gastrointestinal symptoms being the most common. Dyspnea and cough were the most common respiratory complaints. Per the CDC, EVALI remains a diagnosis of exclusion as there is no specific marker or test that exists to confirm the diagnosis. To further complicate the matter, adolescents may not be forthcoming with information about vaping due to fear of repercussions or embarrassment. Negative RVPs helped to narrow the differential diagnosis. Although one patient had a positive RVP result, due to the timing of symptoms and using our clinical judgment, the presentation was thought to be more consistent with EVALI. Physicians therefore need to maintain a high level of suspicion to diagnose EVALI in a timely manner. Mental health issues were the most common comorbidity, and there were no patients who had an active ongoing pulmonary issue. All but one patient either acknowledged vaping marijuana or had a positive urine drug screen for THC, suggesting that marijuana puts one at increased risk for developing EVALI. There was a variable range of vaping duration that did not appear to correlate with severity of lung disease.

Routine chest radiographs were almost always performed, but were often nonspecific and could even be normal, and cannot be used as a reliable tool to evaluate EVALI. We found that the constellation of chest CT findings, which included bilateral ground-glass opacities (most commonly in the dependent portion of lungs), interstitial thickening, and peripheral sparing, was far more diagnostic and helped to exclude other differentials. Pneumomediastinum and consolidation were uncommon. Lab findings were also variable. The most consistent lab results were a significant leukocytosis and elevated inflammatory markers. We found trending inflammatory markers to be helpful in monitoring disease recovery as they correlated with symptoms.

All but one patient required hospitalization for EVALI. The average duration of stay was one week, during which most patients remained on methylprednisolone until discharge, independent of their respiratory or dietary status. Oxygen requirements were variable, with no clear indication of which patients would require more intensive support. Most patients were able to maintain adequate oxygenation on room air or with nasal cannula. Only one patient required intubation, mechanical ventilation, and subsequent ECMO support, making this severe presentation uncommon.

Prior to discharge, spirometry, diffusing capacity for carbon monoxide, and six-minute walk tests were completed if the patient was able. Although many patients were able to maintain adequate oxygen saturations at rest at time of discharge, it was not uncommon for desaturations to occur with activity. This led to four of our patients being discharged home with supplemental oxygen via nasal cannula. The most common abnormality seen on spirometry was mild obstructive lung disease, followed by impaired diffusion. This finding was likely due to the inflammatory effect of vaping in both the airways and lung parenchyma. Psychology consults were common to address mental health comorbidities while inpatient. Only one patient received nicotine replacement while intubated on ECMO support. No patients endorsed nicotine withdrawal symptoms or requested assistance with nicotine replacement. Patients were prescribed, on average, a steroid taper to complete over 14 days after discharge from the hospital.

All patients who followed up reported improved symptoms following steroid treatment and with abstinence from vaping. Based on spirometry results, repeat CT scans, and clinical assessment, it appears that outcomes are favorable.

## Conclusions

Vaping is a serious issue that can cause acute devastating lung injury to our youth. Our experience with adolescent EVALI highlighted the variety of presentations and course of illness among patients. Eliciting a vaping history, negative infectious testing, elevated inflammatory markers, and characteristic CT findings were key to making the diagnosis of EVALI. Research is still limited on adolescent EVALI and the long-term sequala; however, outcomes in our population were reassuring with abstinence and steroids.
